# Metabolic parameters in F-fluorodeoxyglucose positron emission tomography/computed tomography for treatment response in colorectal carcinoma

**DOI:** 10.1590/1806-9282.20241024

**Published:** 2025-09-19

**Authors:** Fatih Güzel, Şadiye Altun Tuzcu, Bekir Taşdemir

**Affiliations:** 1 Dicle University, Faculty of Medical, Department of Nuclear Medicine – Diyarbakır, Turkey.

**Keywords:** Colorectal carcinoma, Medicine, nuclear, Imaging, molecular, CT scanner, X-ray, Positron-emission tomography, Tumor volume, Fluorodeoxyglucose F18

## Abstract

**OBJECTIVE:**

In daily practice, ^18^F-fluorodeoxyglucose positron emission tomography/computed tomography plays a crucial role in the clinical follow-up to evaluate treatment response and detect recurrence in patients with elevated serum tumor markers. In this research, we aimed to elucidate whether there was a correlation between markers and semiquantitative-metabolic parameters.

**METHODS:**

In this study, 66 patients who applied to our institution with a histopathological diagnosis of colorectal carcinoma and had two consecutive ^18^F-fluorodeoxyglucose positron emission tomography/computed tomography scans for diagnosis or response to treatment were enrolled. The obtained images were evaluated based on response evaluation criteria in solid tumors and positron emission tomography response criteria in solid tumors criteria. The patients were divided into two groups: regression patient group (n=45) and progression patient group (n=21).

**RESULTS:**

There was a high positive correlation between whole body-total lesion glycolysis and serum tumor marker (r=0.618, p=0.000), a moderate positive correlation between SUV_max_ (standardized uptake value) and primary tumor-total lesion glycolysis and serum tumor marker (r=0.497, p=0.001; r=0.511, p=0.000), and a weak positive correlation between SUV_mean_ and whole body-metabolic tumor volume and serum tumor marker (r=0.435, p=0.003) in patients achieving regression. There was a moderate positive correlation between whole body-total lesion glycolysis and tumor marker in the progression group (r=0.596, p=0.004) and a weak positive correlation between SUV_max_, SUV_mean_, total lesion glycolysis, whole body-metabolic tumor volume, and tumor marker in the progression group (r=0.343, p=0.128; r=0.299, p=0.188; r=0.279, p=0.220; r=0.374, p=0.095).

**CONCLUSION:**

We concluded that ^18^F-fluorodeoxyglucose positron emission tomography/computed tomography will provide beneficial outcomes in evaluating treatment response and detecting recurrent disease in patients with colorectal carcinoma. In this context, the whole body-total lesion glycolysis value is the most sensitive parameter that can be an alternative to SUV_max_ in predicting treatment response.

## INTRODUCTION

Colorectal cancer (CRC) ranks third among cancers worldwide and constitutes 10% of all cancers. It is the most common type of cancer after prostate and lung cancer in men and breast and lung cancer in women. It is the second most common cause of cancer-related death, and the five-year survival rate is approximately 50–60%. The risk factors for developing colorectal cancers can be elaborated as being over 50 years of age, having a family history of cancer, smoking, the presence of genetic mutations, diet, the presence of polyps, and the presence of inflammatory bowel diseases, especially ulcerative colitis^1,2^.

An anamnesis and detailed physical examination, contrast-enhanced radiological studies, computed tomography (CT), magnetic resonance imaging (MRI), positron emission tomography/computed tomography (PET/CT), proctosigmoidoscopy, flexible sigmoidoscope, colonoscopic imaging and biopsy, and transrectal endoscopic ultrasonography (EUS) are utilized in the diagnosis of colorectal cancer. Serum tumor markers such as carcinoembryonic antigen (CEA) and cancer antigen (CA) 19-9 also contribute greatly to clinical follow-up. In colorectal cancers, ^18^F-FDG PET/CT is an important imaging method in helping to determine tumor localization, primary staging, and determination of biopsy area. It also enables the detection of recurrences and evaluation of the response to treatment^3,4^.

^18^F-FDG PET/CT is more successful than other imaging methods in evaluating the treatment response, especially in the early phase of locally advanced colorectal cancers after neoadjuvant chemoradiotherapy (CRT) and in metastatic cases where local or systemic treatments are used^4^.

Within the scope of this research, we aimed to elucidate whether there was a correlation between markers and semiquantitative-metabolic parameters (SUV_max_, SUV_mean_, metabolic tumor volume [MTV], total lesion glycolysis [TLG]) obtained with ^18^F-fluorodeoxyglucose positron emission tomography/computed tomography (^18^F-FDG PET/CT) and which metabolic parameter was more sensitive in predicting response to treatment.

## METHODS

In this study, 66 patients who applied to the Dicle University Faculty of Medicine Department of Nuclear Medicine between 31 May 2018 and 9 September 2023 with a histopathological diagnosis of colorectal carcinoma and who had two consecutive ^18^F-FDG PET/CT scans taken in our institution for diagnosis or response to treatment were enrolled. The obtained images were evaluated by experienced nuclear medicine specialists based on RECIST and PERCIST criteria. According to the PET/CT evaluation results, we divided our study group into two groups: the regression patient group (n=45) and the progression patient group (n=21).

All procedures followed were in accordance with the ethical standards of the responsible committee on human experimentation (institutional and national) and with the Helsinki Declaration of 1975, as revised in 2008. Ethics committee approval was granted from our institution on 13.09.2023 with protocol number 282, and informed consent was obtained from all participants.

The age, gender, location of the primary tumor (cecum, ascending colon, transverse colon, descending colon, sigmoid, rectum), response to treatment (regression, progression), pre-treatment and post-treatment ^18^F-FDG PET/CT parameters (SUV_max_, SUV_mean_, MTV, and TLG), and any decrease or increase in serum tumor markers (CEA, CA 19-9) obtained during these consecutive imaging periods were interpreted.

Patients with a second malignancy and patients who were operated on for their primary tumor or underwent RT for the primary tumor were excluded from the study in order not to cause bias in the outcomes.

The Siemens Horizon brand PET/CT device with a 3D-time of flight (3D-TOF) feature and a section thickness of 3 mm was used for imaging. The images are created using PET iterative and CT bp-line of response (LOR) reconstruction processing methods. The device was set to 80 mA and 120 kV for low-dose, non-contrast CT images. During the ^18^F-FDG PET/CT imaging periods, CEA and CA 19-9 levels were measured using immunological methods using the Beckman Coulter DxI 600 device. SUV_max_ and SUV_mean_ measurements of colorectal primary tumors were obtained via ^18^F-FDG PET/CT and MTV-TLG measurements were performed manually using volumetric area of interest drawings, with a 40% SUVmax threshold value, separately for the primary tumors and their lymph node, liver, and other visceral metastases, which were considered a positive focus. For all colorectal-located primary tumors, 3D regions of interest were drawn on pre-treatment and post-treatment PET/CT images, and SUV_max_, SUV_mean_, Primary tumor MTV (PT-MTV), and Primary tumor TLG (PT-TLG) measurements were conducted. Separate measurements have been taken for metastatic lymph nodes, liver, and other visceral metastases, MTV, and TLG.

### Statistical analysis

Patient data collected within the scope of the study were analyzed with the IBM Statistical Package for the Social Sciences (SPSS) for Windows 26.0 (IBM Corp., Armonk, NY) package program. Frequency and percentage for categorical data and mean and standard deviation for continuous data were given as descriptive values. It was checked whether the data were normally distributed with the Kolmogorov-Smirnov and Shapiro-Wilk tests. The Wilcoxon Test was used to compare the previous and next groupings that did not show normal distribution. Spearman correlation analysis was performed for the relationship between variables. The results were considered statistically significant when the p-value was less than 0.05.

## RESULTS

In this study, pre-treatment and post-treatment ^18^F-FDG PET/CT images of a total of 66 patients, 35 (53%) men and 31 (47%) women, with an average age of 53.6±14.65 years, were evaluated. Regression was detected in 68% (n=45) of patients, and progression was detected in 32% (n=21) of patients. Thirty-two (48%) of the patients were diagnosed with primary colon cancer, and 34 (52%) were followed up with a diagnosis of rectal cancer.

The study group consisted of 6 patients with the primary tumor, 20 patients with local lymph node involvement in addition to the primary colorectal tumor, 2 patients with only liver involvement in addition to the primary tumor, 18 patients with both lymph node and liver involvement in addition to the primary tumor, and 18 patients with local lymph node involvement in addition to the primary tumor. There were 16 patients with lymph node, liver, and visceral organ involvement. Of the 16 patients with visceral organ involvement, 7 had lung involvement, 6 had bone involvement, 2 had lung+bone involvement, and 1 had pleural involvement. Adenocarcinoma was detected in 63 patients, signet ring cell carcinoma in 1 patient, mucinous carcinoma in 1 patient, and small cell carcinoma in 1 patient.

CEA was used for clinical-laboratory follow-up in 59 patients, and CA 19-9 in 7 patients. The mean tumor marker decrease rate in the patient group with regression was −0.67±0.25 (mean decrease: 67±25%), and the mean tumor marker increase rate in the patient group with progression was 3.05±5.71 (mean increase: 305±521%) (Tables 1 and 2).

**Table 1:** Significance relationship between volumetric parameter decline rates and tumor markers decline rates in the regression patient group.

(n=45)	Mean±SD	Median (min/max)	p-value
SUV_max_ ratio	-0.45±0.24	-0.44 (-0.85/-0.07)	0.001
TM ratio	-0.67±0.25	-0.71 (-0.99/-0.13)	
SUV_mean_ ratio	-0.43±0.23	-0.43 (-0.84/0.31)	0.001
TM ratio	-0.67±0.25	-0.71 (-0.99/-0.13)	
PT-MTV ratio	-0.40±0.47	-0.48 (-0.98/1.85)	0.001
TM ratio	-0.67±0.25	-0.71 (-0.99/-0.13)	
PT-TLG ratio	-0.60±0.44	-0.69 (-0.99/1.42)	0.569*
TM ratio	-0.67±0.25	-0.71 (-0.99/-0.13)	
WB-MTV ratio	-0.49±0.46	-0.55 (-0.98/1.77)	0.005
TM ratio	-0.67±0.25	-0.71 (-0.99/-0.13)	
WB-TLG ratio	-0.68±0.35	-0.75 (-0.98/1.12)	0.296*
TM ratio	-0.67±0.25	-0.71 (-0.99/-0.13)	

Mean: mean; SD: standard deviation; n: number of cases; Med: median; min: minimum; max: maximum; SUV_max_: maximum standard uptake value; SUV_mean_: mean standard uptake value; WB-MTV: whole body-metabolic tumor volume; WB-TLG: whole body-total lesion glycolysis; TM: tumor marker.

* No statistically significant difference was observed.

**Table 2:** Significance relationship between volumetric parameter increase rates and tumor markers increase rates in the progressive patient group.

(n=21)	Mean±SD	Median (min/max)	p-value
SUV_max_ ratio	0.21±0.38	0.22 (-0.46/0.98)	0.001
TM ratio	3.05±5.71	0.86 (-0.13/25.75)	
SUV_mean_ ratio	0.33±0.53	0.24 (-0.56/1.61)	0.002
TM ratio	3.05±5.71	0.86 (-0.13/25.75)	
PT-MTV ratio	3.96±15.45	0.35 (-0.18/71.32)	0.073*
TM ratio	3.05±5.71	0.86 (-0.13/25.75)	
PT-TLG ratio	0.72±0.86	0.55 (-0.37/2.92)	0.027
TM ratio	3.05±5.71	0.86 (-0.13/25.75)	
WB-MTV ratio	1.22±1.18	0.93 (-0.08/4.88)	0.205*
TM ratio	3.05±5.71	0.86 (-0.13/25.75)	
WB-TLG ratio	1.52±2.15	0.87 (-0.18/9.7)	0.434*
TM ratio	3.05±5.71	0.86 (-0.13/25.75)	

Mean: mean; SD: standard deviation; n: number of cases; Med: median; min: minimum; max: maximum; SUV_max_: maximum standard uptake value; SUV_mean_: mean standard uptake value; WB-MTV: whole body-metabolic tumor volume; WB-TLG: whole body-total lesion glycolysis; PT-MTV: primary tumor-metabolic tumor volume; PT-TLG: primary tumor-total lesion glycolysis; TM: tumor marker.

* No statistically significant difference was observed.

There was a statistically significant difference between the decrease rates of SUV_max_, SUV_mean_, PT-MTV value (SUV_max_ ratio, SUV_mean_ ratio, MTV ratio) and whole-body total MTV value (WB-MTV ratio) measured from the primary tumor and tumor marker decline rates (p<0.001, p<0.001, p<0.001, and p=0.005) in patients achieving regression (Table 3).

**Table 3:** Correlation of volumetric parameters with tumor markers in the patient group showing regression and the progressing patient group.

Regression (n=45)		SUV_max_ ratio	SUV_mean_ ratio	MTV ratio	TLG ratio	WB-MTV ratio	WB-TLG ratio
TM ratio	Correlation coefficient (r)	0.497	0.435	0.242	0.511	0.443	0.618*
	p-value	0.001	0.003	0.109	0.001	0.002	0.001
Progression (n=21)		SUV_max_ ratio	SUV_mean_ ratio	MTV ratio	TLG ratio	WB-MTV ratio	WB-TLG ratio
TM ratio	Correlation coefficient (r)	0.343	0.299	0.099	0.279	0.374	0.596*
	p-value	0.128	0.188	0.670	0.220	0.095	0.004

n: number of cases; SUV_max_: maximum standard uptake value; SUV_mean_: mean standard uptake value; MTV: metabolic tumor volume; TLG: total lesion glycolysis; WB-MTV: whole body-metabolic tumor volume; WB-TLG: whole body-total lesion glycolysis; TM: tumor marker.

* A high level of relationship in a positive direction.

A statistically significant difference was found between the SUV_max_, SUV_mean_, and TLG value increase rates (SUV_max_ ratio, SUV_mean_ ratio, and TLG ratio) measured from the primary tumor and the tumor marker increase rates (p=0.001, p=0.002, and p=0.027) in patients showing progression (Table 3).

In patients achieving regression, a high-positive correlation between WB-TLG and serum tumor markers (r=0.618, p=0.000), a moderate positive correlation between SUV_max_ and PT-TLG and serum tumor markers (r=0.497, p=0.001; r=0.511, p<0.001), and a weak positive correlation between SUVmean and WB-MTV and serum tumor markers (r=0.435, p=0.003; r=0.443, p=0.002) were detected. In the patient group with progression, a moderate positive correlation between WB-TLG and serum tumor marker (r=0.596, p=0.004), a weak positive correlation between SUVmax, SUVmean, PT-TLG, WB-MTV, and serum tumor marker (r=0.343, p=0.128; r=0.299, p=0.188; r=0.279, p=0.220; r=0.374, p=0.095). No statistically significant difference was detected in other volumetric parameters.

## DISCUSSION

CRC, one of the most common cancer types worldwide, accounts for 10% of all cancers^1^. According to 2019 WHO data, CRC is the 2nd most common cause of cancer-related death when both genders are taken into account. The incidence and mortality rates of CRC in men are relatively higher than in women^5^. In the Topdagi et al.^6^ study with 752 CRC patients, 56.8% were men, and 43.2% were women. In our study, 53% of the patients were men and 47% were women. The mean age of the patients in our study was 53.59±14.65 years. Patients aged 40 years and over constituted 84.85% of our entire patient group.

In colorectal cancers, ^18^F-FDG PET/CT is useful in staging, evaluating treatment response, and detecting recurrence-metastasis^7^. In PET/CT, visual examination of the images obtained is very important for response to treatment, but semiquantitative (SUV_max_, SUV_mean_, MTV, and TLG) evaluation of the images together with FDG distribution provides an objective approach for the characterization of lesions and evaluation of treatment response. In this context, measurements and changes in SUV values are frequently used in routine practice to evaluate treatment response^8^. The most common use of CEA is detecting recurrent diseases during follow-up. On the other hand, CA 19-9 is associated with evaluating responses to treatment rather than being a diagnostic marker^9-11^.

In this study, when evaluating treatment response, we used serum tumor markers frequently used in routine practice as a basis for comparison in clinical-laboratory response follow-up. For this reason, we excluded patients who did not have elevated serum tumor markers at the time of histopathological diagnosis. We included patients with elevated serum tumor markers at the time of diagnosis and for whom these markers were used during follow-up. In some patients, the elevation of two markers (CEA, CA 19-9) was detected simultaneously, while in others, the elevation of a single marker was detected. Nozoe et al.^12^ found that the simultaneous high levels of CEA and CA 19-9, two of the serum tumor markers in patients before treatment, were associated with poor prognosis in 133 CRC patients. Our study found a progression rate of 21.6% in the ^18^F-FDG PET/CT examination of 37 patients in whom only one of the serum tumor markers was high before treatment. In comparison, we found a progression rate of 21.6% in 29 patients in whom both serum tumor markers (CEA and CA 19-9) were higher simultaneously before treatment. In the patient's ^18^F-FDG PET/CT examination, we detected a progression rate of 44.8%. Thus, we supported the idea that the elevation of two markers present during diagnosis or before treatment indicates a poor prognosis. Studies comparing metabolic parameters and serum tumor markers were performed with a single ^18^F-FDG PET/CT image taken during the diagnosis or follow-up period, and serum tumor markers were examined during the imaging period. On the contrary, we have used two consecutive ^18^F-FDG PET/CT images taken before and after treatment and the data examined during these periods to compare the decrease and increase rates of serum tumor markers. Milardovic et al.^13^ examined the correlation between SUV_max_, MTV, and TLG values, measured separately from the primary tumor and metastases, and serum CEA levels in their study of 100 patients with CRC. SUVmax, MTV, and TLG indicated a positive correlation with CEA, but only the correlation of CEA and TLG was significant (r=0.678, p=0.001).

Caglar et al.^14^ found that semiquantitative parameters obtained from PET/CT denoted a moderate positive correlation with serum CEA levels in 155 CRC patients. They stated that the correlation coefficients for lesion SUV_max_, WB-MTV, and WB-TLG were 0.45, 0.44, and 0.49 (p<0.0001), respectively. Lu et al.^15^ reported that PT-MTV and PT-TLG significantly correlated with tumor burden in non-metastatic CRC patients. In parallel with these studies, we found a high positive correlation between serum tumor markers and WB-TLG, a moderate positive correlation between SUV_max_ and PT-TLG, and a weak correlation between WB-MTV in patients achieving regression. On the other hand, there was a moderate positive correlation between serum tumor markers and WB-TLG and a weak correlation between WB-MTV and serum tumor markers in patients showing progression. Choi et al.^16^ conducted a retrospective analysis of 29 patients and found a moderate correlation between total MTV values and serum CEA levels.

## CONCLUSION

We have evaluated the pre- and post-treatment ^18^F-FDG PET/CT findings of colorectal cancer cases and serum tumor marker levels in both imaging periods. We concluded that the WB-TLG value is the most sensitive parameter and can be an alternative to SUVmax in predicting treatment response. Additionally, ^18^F-FDG PET/CT provided beneficial outcomes in evaluating treatment response and detecting recurrent diseases and recurrence in patients with colorectal carcinoma. Hence, semiquantitative-metabolic parameters obtained from PET/CT may have prognostic value in colorectal cancer. However, studies should be conducted with a large number of cases to understand the prognostic value of metabolic parameters obtained from PET/CT in patients with colorectal cancer.

## Data Availability

The datasets generated and/or analyzed during the current study are available from the corresponding author upon reasonable request.
